# Macrophage migration inhibitory factor in Nodding syndrome

**DOI:** 10.1371/journal.pntd.0009821

**Published:** 2021-10-18

**Authors:** Gil Benedek, Mahmoud Abed El Latif, Keren Miller, Mila Rivkin, Ally Ahmed Ramadhan Lasu, Lul P. Riek, Richard Lako, Shimon Edvardson, Sagit Arbel-Alon, Eithan Galun, Mia Levite

**Affiliations:** 1 Tissue Typing and Immunogenetics Unit, Department of Genetics, Hadassah Medical Organization and Faculty of Medicine, Hebrew University of Jerusalem, Jerusalem, Israel; 2 Goldyne Savad Institute of Gene Therapy, Hadassah Medical Organization and Faculty of Medicine, Hebrew University of Jerusalem, Jerusalem, Israel; 3 Public Health Consultant, Juba, Republic of South Sudan; 4 External Coordination & Research, Ministry of Health, Juba, Republic of South Sudan; 5 Ministry of Health South Sudan, Juba, Republic of South Sudan; 6 Department of Pediatrics, Neurology Unit, Hadassah Hebrew University Hospital, Jerusalem, Israel; 7 Department of Obstetrics and Gynecology, Hadassah Hebrew University Hospital, Jerusalem, Israel; 8 Faculty of Medicine, The Hebrew University of Jerusalem, Israel; Istituto Superiore di Sanità, ITALY

## Abstract

Nodding syndrome (NS) is a catastrophic and enigmatic childhood epilepsy, accompanied by multiple neurological impairments and neuroinflammation. Of all the infectious, environmental and psychological factors associated with NS, the major culprit is Onchocerca Volvulus (Ov)–a parasitic worm transmitted to human by blackflies. NS seems to be an ’Autoimmune Epilepsy’ in light of the recent findings of deleterious autoimmune antibodies to Glutamate receptors and to Leiomodin-I in NS patients. Moreover, we recently found immunogenetic fingerprints in HLA peptide-binding grooves associate with protection or susceptibility to NS. Macrophage migration inhibitory factor (MIF) is an immune-regulatory cytokine playing a central role in modulating innate and adaptive immunity. MIF is also involved in various pathologies: infectious, autoimmune and neurodegenerative diseases, epilepsy and others. Herein, two functional polymorphisms in the *MIF* gene, a −794 CATT_5–8_ microsatellite repeat and a −173 G/C single-nucleotide polymorphism, were assessed in 49 NS patients and 51 healthy controls from South Sudan. We also measured MIF plasma levels in established NS patients and healthy controls. We discovered that the frequency of the high-expression MIF -173C containing genotype was significantly lower in NS patients compared to healthy controls. Interestingly however, MIF plasma levels were significantly elevated in NS patients than in healthy controls. We further demonstrated that the HLA protective and susceptibility associations are dominant over the MIF association with NS. Our findings suggest that MIF might have a dual role in NS. Genetically controlled high-expression MIF genotype is associated with disease protection. However, elevated MIF in the plasma may contribute to the detrimental autoimmunity, neuroinflammation and epilepsy.

## Author summary

Nodding syndrome (NS) is a devastating and enigmatic childhood disease, accompanied by multiple neurological impairments and neuroinflammation. NS is associated with the parasite Onchocerca volvulus (Ov) and other environmental factors. It was shown that NS seems to be an "Autoimmune-Epilepsy". However, it is still not clear why only some children, within the same family, tribe and district, develop this malady in South-Sudan. Recently, we hypothesized that immunogenetic factors might address this question, and indeed demonstrated that few amino acids in human leukocyte antigen (HLA) peptide-binding grooves associate with either protection or susceptibility to NS. In the current study, we evaluate the contribution of macrophage migration inhibitory factor (MIF) to NS pathology. MIF is an immune-regulatory cytokine that is involved in numerous pathologies, which vary from infectious diseases, autoimmunity to neurodegenerative diseases. Immunogenetic analysis of MIF in South-Sudanese NS patients and healthy controls shed new light on the epidemiology of this disease. Our findings suggest that MIF promoter genotypes that are linked with high-protein expression are associated with disease protection. However, in affected patients, with established NS, MIF plasma levels are elevated compared to control subjects and might be involved with autoimmunity and neuroinflammation.

## Introduction

Nodding syndrome (NS) is an epileptic disease that affects children within the age range of 3 to 15 years. NS is characterized by severe attacks of head nodding, progressive cognitive dysfunction, neurological deterioration, stunted growth and additional pathological neurological features [1–8]. Many of the NS patients also have frequent episodes of another type of seizures: Generalized Clonic Tonic Seizures (GCTS) [7,9].

Various studies described several etiological factors that might contribute to the onset of this syndrome [2,3,10,11,12,13]. The most compelling data point to the association of NS with infection by the parasitic worm Onchocerca Volvulus (OV), transmitted to humans by female blackflies [1,5,8,9,14,15,16,17]. NS was documented mainly in South Sudan, Uganda, and Tanzania. However, it was also reported in other Onchocerciasis endemic regions [1,7,9,16,17]. In fact, Ov infection might induce a broad spectrum of pathologies, termed together Onchocerciasis. Among these Ov-associated pathologies are epileptic seizures, and this kind of epilepsy has been termed Onchocerciasis Associated Epilepsy (OAE) [8,9,15,18,19]. Yet, there is still only limited evidence that the parasite itself invades the CNS.

Another important issue to note is that the most common reported diagnostic method of past or present Ov infection relies on Onchocerciasis skin manifestations and sometime also on the detection of Ov antibodies, and not on direct detection (by PCR) of Ov itself.

While OV seems to be strongly associated with NS onset, there are additional factors that contribute to the entire pathological scenario of NS.

Recently, it was demonstrated by two studies that the pathology of NS could stem from autoimmunity: **1.** Johnson et al. discovered that some NS patients have autoimmune antibodies that recognize both an Ov antigen and a self-peptide of Leiomodin-I. These cross-reactive anti-Leiomodin-I/Ov antibodies were detected in 52% of NS patients, as well as in 30.9% of unaffected controls from the same villages, and were demonstrated to be neurotoxic [20]. **2.** In a very recently published paper by ourselves [21], we studied 30 South Sudanese NS patients and healthy control subjects, and revealed autoimmune antibodies to 3 extracellular peptides of glutamate receptors (GluR) in NS patients: AMPA-GluR3**B**-peptide antibodies (86%), NMDA-NR1-peptide antibodies (77%) and NMDA-NR2-peptide antibodies (87%). Of these 3 GluR antibodies, NS patient’s affinity-purified AMPA-GluR3**B** were found to have on their own tantalizing pathological effects in vitro: they bind, induce rapid production of ROS in, and then kill both human neural cells and human immune T cells within 1 hour only. Moreover, and most important: In vivo, when NS patient’s purified IgG antibodies were released continuously in the brains of normal mice for 1 week, they bound to several brain regions, and induced epileptic seizures and multiple brain damaged, alike documented in brains of NS patients.

Nevertheless, the above findings do not explain why NS affects only some of the children in the very same affected zones, tribes and even families, while other remain healthy, despite the fact they were all most probably exposed to the same environmental factors and infectious organisms. For trying to answer this question, we performed a study aiming to explore whether differences in HLA may be responsible for the intra-individual inter-individual variabilities associated with NS. Excitingly, we discovered that immunogenetics might indeed explain the differences in diseases susceptibility or protection. Specifically, we revealed that NS associates significantly with both protective HLA haplotype: HLA-B*42:01, C*17:01, DRB1*03:02, DQB1*04:02 and DQA1*04:01, and susceptible motif: Ala24, Glu63 and Phe67, in the HLA-B peptide-binding groove. These findings suggest that immunogenetic fingerprints in HLA peptide-binding grooves tentatively associate with protection or susceptibility to NS [22].

However, it is clear that there are additional immunogenetic factors that contribute to disease susceptibility. One of the factors we suspect to be involved is macrophage migration inhibitory factor (MIF)—an immunoregulatory cytokine that is secreted from various cell types in different tissues. It was previously suggested that MIF is an "immunological enabler" which contributes to the inflammatory process by promoting leukocyte recruitment, inhibiting apoptosis of activated monocytes, and enhancing the secretion of proinflammatory cytokines [23–27]. In addition, chronically high MIF levels were shown to override the endogenous and exogenous glucocorticoids (GC) anti-inflammatory effects [28–31].

MIF was also shown to affect pathologies, caused by infection of different parasites, such as bacteria, protozoa and viruses [32–36]. In addition, MIF was reported to have a critical role in various autoimmune diseases, such as multiple sclerosis (MS), rheumatic arthritis (RA) and autoimmune hepatitis [37–41]. In some autoimmune diseases, MIF is a susceptibility factor, while in others MIF is involved mainly in disease progression. Finally, MIF is expressed in the Central Nervous System (CNS) and contributes to the ongoing function of the healthy brain. However, elevated MIF seems to play a detrimental role in neurodegenerative pathologies, among them Epilepsy, traumatic brain injury (TBI), Parkinson’s disease, and Amyotrophic Lateral Sclerosis (ALS) [42–45]. Furthermore, MIF was shown to be a susceptibility gene for autism spectrum disorder, which might developed due to a pre-natal inflammatory event [46]. Taken together, these data indicate that MIF could contribute to NS.

Based on all of the above, we decided to test for MIF in NS patients, and reasoned that there are three routes that MIF might be involved in NS:

**1.** The essential and beneficial contribution of MIF in physiological levels, to the crucial anti-infection immune responses to the Ov parasitic worm, and to other infectious organisms reported in subjects in NS-affected zones, **2.** The detrimental contribution of elevated MIF to the neurotoxic autoimmunity in NS, directed against Glutamate receptors [21] and Leiomodin-I [20] **3**. The direct detrimental contribution of elevated MIF to the epileptic seizures: both to the nodding and to the GCTS, as well as to the additional brain damage, impaired brain function and mental retardation.

MIF expression is regulated by both genetic and epigenetic factors. Two functional polymorphisms located in the *MIF* gene have been reported to modulate *MIF* promoter activity, and to correlate with MIF expression levels: the 794 CATT5-8 microsatellite repeat and the -173 G/C single nucleotide polymorphism (SNP). Increased *MIF* promoter activity is proportional to increased numbers of the CATT repeats at position -794. Furthermore, the CATT7 allele is in strong linkage disequilibrium with the -173 C allele [26,35,36,47,48].

In order to determine whether MIF polymorphism is associated with NS susceptibility, we analyzed *MIF* -173 G/C SNP and MIF -794 CATT repeats in NS patients and healthy controls (i.e. those not affected by NS) from South-Sudan. In addition, we measured MIF plasma levels in the NS patients and healthy controls. Our findings show that high-expression MIF -173C containing genotypes are associated with protection from NS. However, we found that NS patients have elevated MIF plasma levels compared with control subjects, and these findings suggest MIF increased plasma levels might contribute to the autoimmunity on the one hand and to the epilepsy, brain damage and mental retardation on the other.

## Methods

### Ethics statement

The study was authorized by an IRB approval by the Ministry of Health of South-Sudan, related research approval letters, and informed consents of all the NS patients and healthy subjects. In addition, a parent or guardian of study participant provide informed consent on the child’s behalf. A formal written inform consent was obtained.

### Study participants [22]

The study was performed on South Sudanese NS patients (49) (Table 1) and healthy control subjects not affected by NS (51), from the town of Mundri and surrounding villages (a 250 km range area), in Western Equatoria, South-Sudan. The NS patients were of the Moro and Nyangora tribes.

**Table 1:** Clinical characteristics of the South-Sudanese NS patients and healthy controls included in this study [22].

	NS Patients (n = 49)	Healthy Controls (n = 51)
**Gender**		
Female/male (% females)	23/26 (47)	38/13 (75)
**Age at recruitment**		
Mean (years) ± S.D	14.3±4.0	14.8±5.9
Range	4–25	4–25
**Age at initial NS symptoms**		
Mean (years) ± S.D	8.0±2.9	
Range	4–14	
Nodding with food	37/49	
Nodding with other triggers	11/49	
Other seizures	36/49	
Cognitive decline	38/49	
Low muscle mass	31/34	
Wasting	31/34	

### Nodding syndrome diagnosis

Through a prior mobilization drive by the Mundri health workers, community members were requested to identify and bring to Mundri health center **suspected nodding disease cases**, defined at the community level as repetitive, involuntary drops of the head to the chest, with drooling of saliva of a previously normal person.

A pediatric neurologist then elicited history and performed full neurological examination on each **suspected case** for probable nodding syndrome using the **2 major criteria** of **age** (3–15 years) and observed **frequency of nodding** per minute (5–20 nods/minutes). In addition, more comprehensive clinical evaluation was performed for some of them (Table 1). Most of these NS patients had also Generalized Clonic Tonic Seizures (GCTC), on top of the nodding attacks, as well as various additional pathological symptoms. Healthy South Sudanese subjects, at similar age range, and geographical locations to that of the NS patients, were recruited as controls.

Small volume of blood was drawn from all the study subjects by the Sudanese clinicians, and all samples were shipped to Israel. All the studies described in this manuscript were performed in Israel.

Final important note: to the best of our knowledge, the NS patients and the healthy controls whose blood was investigated in this study were NOT tested for Ov infection before the withdrawal of blood for this study. Moreover, we did NOT receive any later information (with regards to Ov infection or any other factor), update, follow-up or blood again, from the same patients and healthy subject since the onset of the study.

### MIF-173G/C genotyping

High molecular weight DNA was extracted from blood samples using the MagNA Pure Lc DNA isolation kit. with the automatic device MagNA Pure (Roche).

A 366-bp fragment of DNA containing the MIF −173 polymorphism (rs755622) was amplified as described before [49]. Briefly, PCR was performed using 200 ng of DNA, 0.5 mM of primers: forward primer (5’-ACTAAGAAAGACCCGAGGC-3’), reverse primer (5’-GGGGCACGTTGGTGTTTAC-3’), and REDExtract-N-Amp PCR Ready Mix (#R4775; Sigma Aldrich). The PCR product was digested overnight using AluI restriction site and was cut into two fragments resulting in a 98- and a 268-bp band, while the C allele contains two AluI restriction sites and was cut into three fragments, resulting in 205-, 98-, and 63-bp bands.

### MIF –794 CATT5–8 genotyping

The MIF -794 CATT5-8 microsatellite was analyzed by the methodology described by Sreih at al. [50]. Briefly, MIF -794 CATT5-8 genotyping was carried out by PCR using a forward primer (5′- GCAGGAACCAATACCCATAGG-3′) and a FAM fluorescent reverse primer (FAM 5′-AATGGTAAACTCGGGGGAC-3′). Automated capillary electrophoresis on a DNA sequencer was performed on each sample and the -794 CATT5-8 repeat length was identified using Genotyper 3.2 software (Applied Biosystems). Although at least eight additional polymorphisms have been identified within the human MIF locus, these additional variants (all SNPs) are rare and have a low likelihood of functionality given their location in introns or within the 3′UTR [47].

### Analysis of plasma MIF levels by ELISA

Plasma MIF concentration was measured by the human MIF Quantikine ELISA kit (DMF008, R&D systems) according to the manufacturer’s instructions. The minimum detectable dose of human MIF using this kit ranges from 0.005–0.068 ng/ml (mean = 0.016ng/ml).

### Statistical analysis

For MIF polymorphisms, Pearson’s χ2 test was used to analyze Hardy–Weinberg equilibrium. Allele and genotype frequencies were compared using Pearson’s χ2. MIF plasma levels were compared using student’s T-test. Bivariate logistic regression analysis was performed using SAS software (version 9.04). Tests with p≤0.05 were considered significant.

## Results

### NS patients and healthy subjects included in the study

A total of 100 South Sudanese children and young adults: 49 NS patients and 51 healthy controls, aged 4 to 25 years, were recruited for the study, most of them from the same Mundri region, and belonging to the Moro and Nyangora tribes, with an approval of the South Sudanese Ministry of Health National IRB. The mean age at initial symptoms was 8 years (SD±2.9). The clinical characteristics and demographic data of the NS patients included in the study are presented in Table 1. Most of the NS patients had GTCS (75%), cognitive deskinment (79%), low muscle mass (91%) and wasting (91%) on top of the nodding attacks. Nodding was frequently triggered by food consumption, immediately following swallowing (77%). Physical examination of NS patients by a pediatric neurologist (S.E. author herein) did not reveal specific ocular or cutaneous stigmata associated with OV. Despite the absence of such signs, it should be pointed out that, eye-examination did not include funduscopy, and that the general health and living-conditions of the population were such that variety infectious diseases were prevalent. Although it was reported that the Mundri region is known to be affected by OV [16,51,52], we cannot conclude whether the specific NS patients whose blood we studied, were infected by Ov. Of note, the NS patients and healthy controls studied inhere were the same subjects that were studied in our previous immunogenetic study [22], except for one added patient.

### MIF promoter polymorphism is associated with NS

The prevalence of the MIF -173 G/C SNP and -794 CATT5-8 microsatellite polymorphism was evaluated in 49 NS patients and 51 healthy controls from South-Sudan. First, we tested the Hardy- Weinberg equilibrium to evaluate if these genetic variations remain constant from generation to generation, in the absence of disturbance by outside factors. No deviation from Hardy-Weinberg equilibrium was detected in the control group (CATT5-8 p = 0.43, G/C p = 0.86). In addition, in the control group the linkage disequilibrium (LD) value between -173C allele and the CATT 7 was D’ = 0.88.

The frequencies of the -173 G/C SNP genotypes are shown in Table 2. The frequencies of the high-expression MIF -173 C containing genotypes (CC/CG), which have been previously shown to be associated with elevated levels of circulating MIF, were significantly lower in the NS group compared with controls (55.1% vs. 78.5%, p = 0.01, respectively). The frequency of the C allele was significantly lower in NS patients (38.7% vs. 53.0, p = 0.04).

**Table 2:** MIF -173 G/C polymorphism in NS patients and healthy controls.

MIF -173 SNP genotype	NS Patients (%) (n = 49)	Healthy Controls (%) (n = 51)	P value	OR (95% CI)
**GG**	22 (44.9)	11 (21.5)		
**GC**	16 (32.6)	26 (50.1)		
**CC**	11 (22.5)	14 (27.4)		
**-173 C containing genotypes**	27 (55.1)	40 (78.5)	0.01	0.33 (0.14–0.80)
**Allele**				
**G**	60 (61.3)	48 (47.0)	0.04	0.56 (0.32–0.98)
**C**	38 (38.7)	54 (53.0)		

P values were calculated by Pearson’s χ2. OR-odds ratio. CI- confidence interval.

The frequencies of the -794 CATT repeat genotypes are presented in Table 3. The frequencies of the high-expression CATT7 containing genotypes were lower in NS patients compared with HC (30.6% vs. 35.3%), but this difference was not significant, perhaps due to the limited sample size. An analysis of inferred haplotypes showed a similar trend with lower frequencies of the combined high-expression genotypes (-173 GC/CC and -794 CATT 6X/7X/8X) in NS patients compared with HC (44.8% vs. 58.8%). Taken together, these set of genetic findings show the genetically control low MIF expression associates NS susceptibility.

**Table 3:** MIF CATT5-8 polymorphism in NS patients and healthy controls.

MIF -794 CATT genotypes	NS Patients (%) (n = 49)	Healthy Controls (%) (n = 51)	P value
**55**	8 (16.3)	10 (19.6)	#
**56**	17 (34.7)	12 (23.5)	#
**57**	2 (4.1)	8 (15.7)	#
**66**	9 (18.4)	11 (21.6)	#
**67**	11 (22.4)	9 (17.6)	#
**68**	1 (2.0)	0	#
**77**	1 (2.0)	1 (2.0)	#
**-794 CATT7 containing genotypes**	15 (30.6)	18 (35.3)	#
**Allele**			
**5**	35 (35.7)	40 (39.2)	#
**6**	47 (47.9)	43 (42.2)	#
**7**	15 (15.3)	19 (18.6)	#
**8**	1 (1.1)	0	#

#- not significant

### HLA and MIF genotypes combined associations with NS

Next, we sought to evaluate the combined contribution of the HLA and MIF genotypes to diseases susceptibility or protection. We demonstrated previously, that the HLA haplotype- HLA-B*42:01, C*17:01, DRB1*03:02, DQB1*04:02 and DQA1*04:01 has a strongest protective effect, since none of the patients carry this haplotype [22]. As presented in S1 Table, in NS patients and healthy controls that do not carry this protective haplotype, the frequency of the high-expression MIF -173C containing genotypes (CC/CG) was still significantly lower in NS patients compared with healthy controls (54.1% vs. 80%, OR = 0.295, p = 0.01, respectively). We further evaluated the relationship of the susceptible motif: Ala24, Glu63 and Phe67, in the HLA-B peptide-binding groove and the MIF -173 G/C genotypes with NS susceptibility using a logistic regression model. Interestingly, while subjects that carry the MIF-173 GG genotype have similar chances to suffer from NS, regardless if they also carry this HLA-B binding groove motif or do not carry this motif. However, subjects that carry the MIF -173 C containing genotypes and the Ala24, Glu63 and Phe67 motif, in the HLA-B peptide-binding groove have more than 8 times chances to suffer from NS compared to subjects that carry only the MIF -173 C contacting genotypes and a different motif in these positions (Table 4).

**Table 4:** Bivariate analysis of HLA and MIF genotypes contribution to disease susceptibility.

Carrier of MIF -173C allele	Carrier of Ala24, Glu63 and Phe67 motif in the HLA-B	NS Patients (%) (n = 48)	Healthy Controls (%) (n = 51)	Odds Ratio Controlled for carrier of MIF -173C allele (95% CI)
No	No	15 (68.2)	7 (31.8)	0.816 (0.17–3.73)
	Yes	7 (63.6)	4 (36.4)	
Yes	No	12 (25.5)	35 (74.5)	8.166 (2.42–27.48)
	Yes	14 (73.7)	5 (26.3)	

CI- confidence interval.

### MIF plasma levels

**Fig 1:**
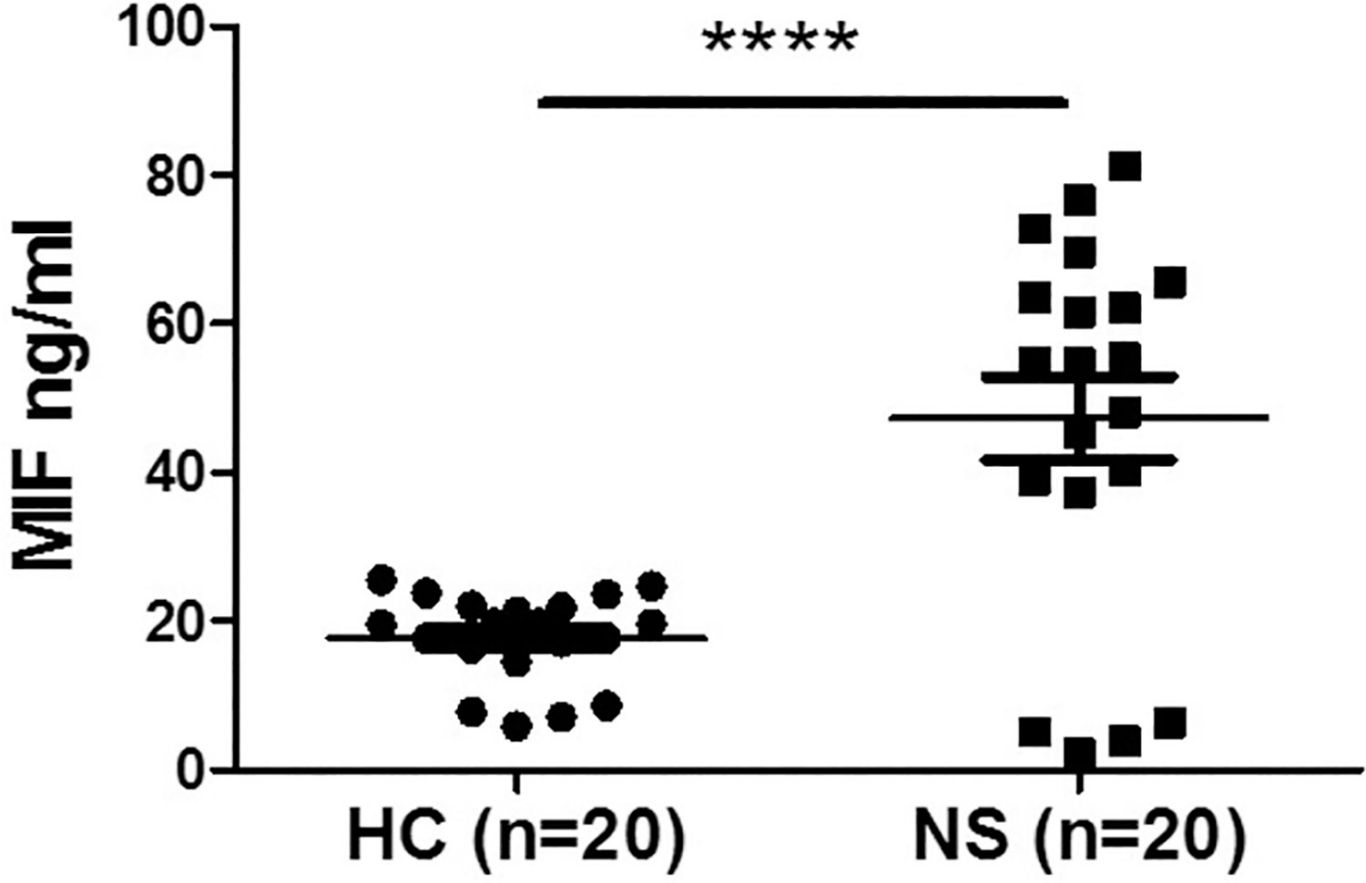
Plasma levels of MIF in NS and healthy controls. MIF concentrations were evaluated by ELISA in healthy controls (n = 20) and NS (n = 20) subject. Data are presented as mean ±SEM, analyzed for significant difference by student’s t-test. **** p<0.0001.

Since elevated MIF levels in the plasma correlate with elevated inflammation, we evaluated circulating MIF levels in plasma samples of 20 NS patients and 20 HC from South-Sudan. We discovered that the mean MIF levels were significantly higher (~ 2.6-fold) in the plasma of NS patients, compared with healthy controls (47.3±25.0 ng/ml vs. 17.8±6.0 ng/ml, ****p<0.0001) (Fig 1). These findings show that circulating MIF in plasma is elevated in patients with NS and suggest they may contribute to the disease.

## Discussion

NS is a devastating childhood neurological disorder, which is characterized by pathological nodding seizures and multiple additional brain pathologies [2,3,4,8,9,21]. The affected children experience a complete and permanent stunting of growth and mental handicap [2,3,4,7,8]. While NS is still enigmatic, it is strongly associated with the parasitic worm OV [1,5,8,9,15,16,17,18,53]. Recently, it was shown that autoimmunity might drive the pathology of this disease [20,21]. In our recent paper we discovered that NS patients’ glutamate receptor antibodies by themselves bind, induce ROS in, and kill both neural cells and T cells in vitro. Moreover, in vivo, NS patient’s IgG that was released in the brain of normal mice for one week, induced all the following detrimental effects: 1. Seizures, 2. Cerebellar Purkinje cell loss, 3. Degeneration in the hippocampus and cerebral cortex, and 4. Elevation of CD3+ T cells, and of activated Mac-2^+^ microglia and GFAP^+^ astrocytes in both the gray and white matter of the cerebral cortex, hippocampus, corpus colosseum and cerebellum of mice [21].

In yet another very recent immunogenetic study, we recently discovered that NS associates significantly with both protective HLA haplotype: HLA-B*42:01, C*17:01, DRB1*03:02, DQB1*04:02 and DQA1*04:01, and susceptible motif: Ala24, Glu63 and Phe67, in the HLA-B peptide-binding groove. These amino acids create a hydrophobic and sterically closed peptide-binding HLA pocket, favoring proline residue [22].

Herein, we discovered that another immunogenetic factor- MIF is associated with NS. The genetic findings in the current study add to the immunogenetic fingerprints we revealed in our study on HLA in NS study [22], and may explain why under the same environmental factors, only some children, within the same families, tribes and districts, develop this malady while others can prevent or overcome it and therefore remain healthy. Strikingly, we discovered that the HLA haplotype HLA-B*42:01, C*17:01, DRB1*03:02, DQB1*04:02 and DQA1*04:01 has a dominant protective effect over the MIF -173 CC/CG genotypes. The later was still significantly associated with disease protection in subjects that did not carry this HLA haplotype. Furthermore, the presence of the HLA-B binding groove motif (Ala24, Glu63 and Phe67) associated with disease susceptibility in subjects that carry the MIF -173 CC/CG genotypes. Suggesting that the HLA susceptibility or protective effects are stronger than the MIF effect in NS. Nonetheless, the MIF -173C allele was found to be significantly associated with disease protection in South-Sudanese.

In the current study, we studied both DNA and serum samples of NS patients and healthy controls from the same region in South-Sudan. The MIF promoter genotype frequencies of the South Sudanese healthy control population were in concordance with the frequencies reported by Zhong et al. for healthy Africans and with similar LD value between the -173 C allele and the CATT7 [48]. In contrast to Caucasians, in Africans the frequency of the -173 C allele was found to be higher than the -173 G allele. This was not the case for NS patients we studied, that were found herein to exhibit significantly lower frequencies of the high-expression MIF genotypes.

In this study, we made another novel discovery: the circulating MIF plasma levels are significantly elevated- more than two-fold higher—in NS patients compared with healthy controls.

**Fig 2:**
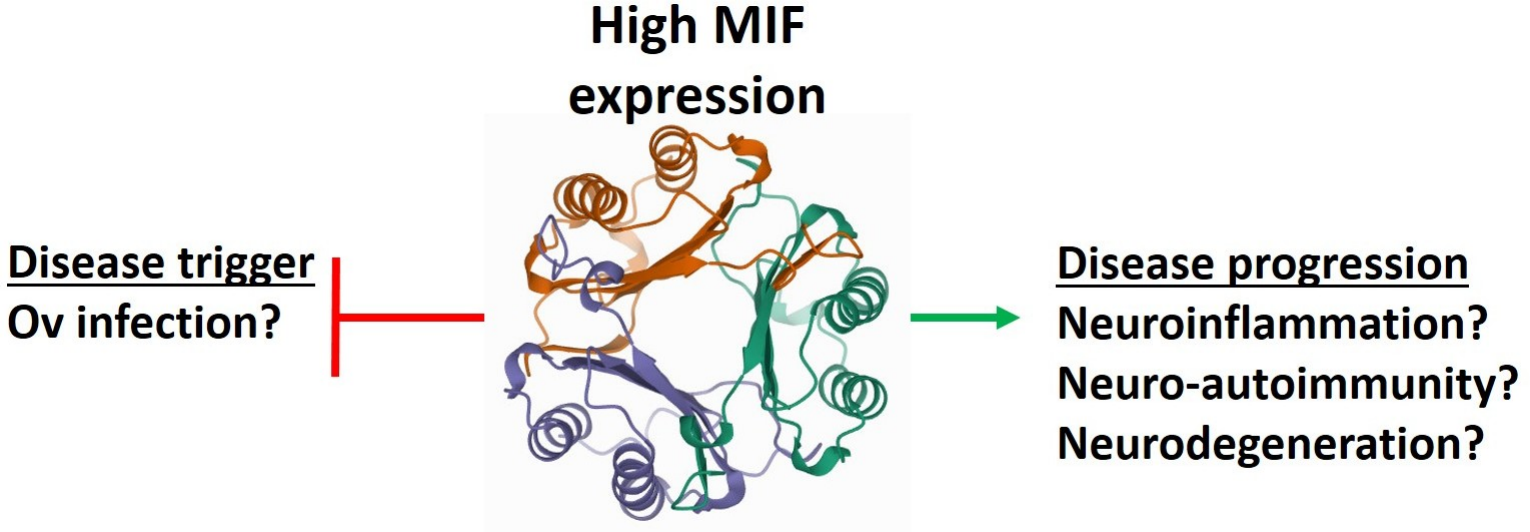
Hypothesized MIF dual role in NS. Genetically controlled basal levels of MIF are associated with NS protection. During NS progression, elevated MIF serum levels might be associated with neuroinflammation, neuro-autoimmunity and possibly neurodegeneration. MIF is illustrated by a three-color trimer.

Based on both their genetic and the protein findings with regards to MIF in NS, we raise herein a novel hypothesis: that MIF might have a dual role in NS pathology, in different time points. We propose that on the one hand the genetically controlled physiological MIF levels confer NS protection. We further propose that in a later time point along the scenario of the disease, once NS has been established, MIF is produced and secreted in high levels and contributes to the development and progression of both autoimmunity, neuroinflammation, subsequent epilepsy and additional profound brain damage. This hypothesis is presented in Fig 2.

Previously Sreih et al. demonstrated such dual effect of MIF in SLE, where on one hand high-expression MIF genotype was associated with disease protection but on the other hand, in patients with established SLE, high-expression MIF genotype was associated with disease progression and end-organ damage [50].

A similar role of MIF was also observed in Tuberculosis, in which high-expression MIF allele confer disease protection whereas circulating MIF that were found to be elevated in patients with active pulmonary tuberculosis, seem to contribute to disease progression [54,55].

This dichotomic role of MIF could also be attributed to its unique properties that enable it to act both extracellularly and intracellularly [56–58]. The extracellular activities are induced by binding of MIF to its cell surface receptors (CD74, CXCR2, CXCR4 and CXCR7) and could result, for example, in chemotactic cell migration [25]. On the other hand, cytosolic MIF was shown to bind to JAB1 and affect AP-1 transcription factor activities [57]. In addition, MIF has an intracellular catalytic thiol-protein oxidoreductase activity and a tautomerase activity (the role of the later in mammalians is not completely clear) [59,60]. Interestingly, it was reported that some of the intracellular activities of MIF take place in neurons: 1. MIF acts as chaperon that binds mutant SOD1 and inhibits the accumulation of misfolded SOD1 [43,61]. 2. MIF is an intracellular inhibitor of Angiotensin II induced activation of neurons [62].

These activities of MIF also might explain MIF dual role in NS: The extracellular activity of MIF affects disease protection from an unknown trigger (possibly Ov infection), whereas, both intracellular and extracellular activities of MIF could affect the inflammatory/autoimmune process and/or the neurodegenerative process in NS.

Taken together we foresee that MIF could affect NS on three different levels, and most probably at three different time points: 1. Assuming that OV infection is one of the major risk factors, physiological and essential MIF might be involved in the protective immunity against this parasite. If this is indeed the case, individuals that produce low physiological levels of MIF, due to their genetic background, would be more susceptible to develop NS; 2. Elevated and detrimental MIF levels are secreted later, in response to chronic ongoing Ov infection and autoimmunity in NS patients and contribute to the perpetuation and severity of the infection, inflammation and/or autoimmunity. 3. Elevated and detrimental MIF levels have direct detrimental effects on the brain, and contribute pathologically to the development of epileptic seizures, and to the additional brain damage and cognitive impairments that characterize NS.

MIF is a key mediator of various inflammatory diseases that varies form autoimmune diseases such as Rheumatoid Arthritis (RA), Systemic Sclerosis (SS), Systemic Lupus Erythematosus (SLE) and Multiple Sclerosis (MS), to infectious diseases such as Malaria, Tuberculosis, Leishmania and others [24,38,39,40,41,50,54,55,63,64,65]. Filbey at el. demonstrated that MIF is critical for type 2 effector cell immunity to helminths [34]. In parasitic infection, MIF is essential in activation of polymorphonuclear cells (PMN), such as neutrophils and eosinophils, and alternatively activated macrophages (AAM). Various studies demonstrated elevated parasitic burden in MIF deficient mice compared with wild type mice [32,33,34,54,66,67].

Of interest, filarial parasite, such as OV, secrete MIF homologue proteins that are able to modify the immune response to enable their survival [66,68,69,70]. These findings, pose a paradox: why would parasites secrete an inflammatory mediator that is able to amplify the immune response?

A possible explanation is that the induction of alternatively activated immune response by parasites is aimed to interfere with full T cell activation, in order to maintain a delicate balance between complete parasitic clearances that can cause devastating tissue damage and parasitic tolerance [71,72]. Usually, helminth parasites cause an asymptomatic infection that is characterized by AAM and down-regulated immune response [73,74]. We propose that in NS patients, which might be infected by OV, this balance has been broken, and the elevated levels of human MIF might contribute both to the uncontrolled amplification of the inflammatory in the periphery, and to the neuroinflammatory response in the brain, which leads to the devastating symptoms of the disease.

NS is a neurological disorder. In this regard, MIF was shown to affect the development and function of various cell types in the CNS, such as astrocytes, microglia and neurons. In addition, MIF is involved in different neurodegenerative diseases, including epilepsy [37,42,43,44,45,75,76]. For example, Quantitative real-time PCR (qPCR) revealed that MIF transcript gradually increases in brain during development. Furthermore, immunofluorescence histochemistry in brain regions revealed MIF-immunoreactive cells within the entire depth of the developed neocortex [76]. A further recent study potentially relevant to NS revealed that CSF levels of MIF are significantly higher in children suffering from acute encephalopathy and having poor-prognosis, than in good-prognosis patients, and suggest that MIF may be markers of poor prognosis [77]. In addition, MIF deficient mice demonstrated behavioral changes compared to normal wild type (WT) mice that resulted in anxiety depression and memory loss [78]. Although we did not evaluate MIF levels in the CNS, it is plausible that MIF CNS levels are also increased in the brains of NS patients, as we found herein in the blood, and that such elevated levels might contribute directly to the epileptic seizures.

As in our previous NS studies [21,22], two issues regarding the cohort size and the geographical zone of the patients and controls, are noteworthy.

First, that our findings call for, and hopefully will stimulate, larger scale immunogenetic, autoimmune, inflammatory and neurological studies on many more NS patients in different countries, and on other forms of OAE, as well as in NS animal models.

Second, we had great difficulties in recruiting South Sudanese NS patients and healthy subjects to this study, due to several reasons, primarily the active ongoing war. As such, recruiting 49 NS patients and 51 healthy subjects was an achievement, although the study could benefit from more.

Taken together, our current and previous studies suggest that NS is a complex multi-facet disease. We demonstrated that in this NS, immunogenetic fingerprints of an individual that may lead to either susceptibility or protection from the initial disease trigger. In a later stage, these factors might also affect the subsequent pathological chronic inflammation, autoimmunity and neurological symptoms.

## Supporting information

S1 Table MIF -173 G/C polymorphism in NS patients and healthy controls that do not carry the protective HLA haplotype. (DOCX)
