# Supplementary material for: Macrophage migration inhibitory factor in Nodding syndrome
Source: PLoS Negl Trop Dis. 2021 Oct 18;15(10):e0009821. doi: 10.1371/journal.pntd.0009821 (PMC8553141; doi:10.1371/journal.pntd.0009821)
Supplement: S1 Table — (DOCX) [file pntd.0009821.s001.docx]

| **OR (95% CI)** | **P value** | **Healthy Controls (%) (n=40)** | **NS Patients (%) (n=48)** | **MIF -173 SNP genotype** |
| --- | --- | --- | --- | --- |
|  |  | 8 (20) | 22 (45.8) | **GG** |
| 0.29  (0.11-0.77) | 0.01 | 32 (80) | 26 (54.2) | **-173 C containing genotypes** |

**S1 Table: MIF -173 G/C polymorphism in NS patients and healthy controls that do not carry the protective HLA haplotype**

P values were calculated by Pearson’s χ2. OR-odds ratio. CI- confidence interval.
